# *CCNE1* and *BRD4* co-amplification in high-grade serous ovarian cancer is associated with poor clinical outcomes

**DOI:** 10.1016/j.ygyno.2020.01.038

**Published:** 2020-02-07

**Authors:** Shariska Petersen, Andrew J. Wilson, Jeff Hirst, Katherine F. Roby, Oluwole Fadare, Marta A. Crispens, Alicia Beeghly-Fadiel, Dineo Khabele

**Affiliations:** a Division of Gynecologic Oncology, Department of Obstetrics & Gynecology, The University of Kansas Medical Center, Kansas City, KS, United States of America; b Department of Obstetrics & Gynecology, Vanderbilt University Medical Center, Nashville, TN, United States of America; c Department of Anatomy & Cell Biology, University of Kansas Medical Center, Kansas City, Kansas, United States of America; d Department of Pathology, University of San Diego, San Diego, CA, United States of America; e Vanderbilt-Ingram Cancer Center, Nashville, TN, United States of America; f Division of Epidemiology, Department of Medicine, Vanderbilt University Medical Center, Nashville, TN, United States of America; g The University of Kansas Cancer Center, Kansas City, KS, United States of America

**Keywords:** Homologous recombination proficient, High-grade serous ovarian cancer, CCNE1, Cyclin E, BRD4

## Abstract

**Objective.:**

High-grade serous ovarian cancer (HGSOC) is the most common and lethal histological subtype of epithelial ovarian cancer. HGSOC with cyclin E1 gene (*CCNE1*) amplification and bromodomain and extraterminal 4 (*BRD4*) amplification have been associated with poor outcomes. Our objective was to evaluate clinical outcomes of HGSOC with co-amplification of *CCNE1* and *BRD4* and high protein expression of cyclin E and BRD4.

**Methods.:**

Copy number amplification data were extracted from The Cancer Genome Atlas (TCGA) for 579 HGSOC. Reverse phase protein array (RPPA) TCGA data were used to determine cyclin E and BRD4 protein expression in 482 HGSOC. Cyclin E and BRD4 protein expression by immunohistochemistry (IHC) was evaluated in a tissue microarray (TMA) of 110 HGSOC. Measured clinical outcomes were survival and platinum sensitivity.

**Results.:**

Of 30% of HGSOC with amplifications in *CCNE1* or *BRD4*, 8% have both *CCNE1* and *BRD4* amplification. Protein expression of cyclin E and BRD4 are positively correlated, both by RPPA (r = 0.23; *p* < 0.001) and by IHC (r = 0.21; *p* = 0.025). Patients with *CCNE1* and *BRD4* co-amplified HGSOC have worse overall survival than patients without amplifications, 39.94 vs 48.06 months (*p* = 0.029). High protein expression of cyclin E, but not BRD4, was associated with poor overall survival (HR 1.62, 1.04–2.53, *p* = 0.033) and platinum resistance (*p* = 0.016).

**Conclusion.:**

HGSOC with *CCNE1* and *BRD4* co-amplification are associated with poor overall survival. Further studies are warranted to determine the use of protein expression by IHC as a surrogate marker for *CCNE1* and *BRD4* co-amplified HGSOC.

## 1. Introduction

Epithelial ovarian cancer (EOC) is the fifth most common cause of cancer mortality among women in the United States [1]. High-grade serous ovarian cancer (HGSOC) is the most common and lethal histological subtype, accounting for approximately 60% of cases. Most women (>60%), are diagnosed with advanced stage III/IV disease. Standard of care for advanced staged disease includes radical tumor debulking surgery and platinum-based chemotherapy. Poly ADP ribose polymerase inhibitors (PARPi) are FDA-approved drugs that significantly improve clinical outcomes, especially in women with germline *BRCA1/2* mutations [2–9]. Tumors with *BRCA1/2* mutations are, by definition, homologous recombination (HR) deficient. PARPi induce synthetic lethality in *BRCA1/2* mutated tumors by inhibiting repair of single strand DNA breaks, resulting in double strand breaks that require the HR pathway for efficient DNA repair [10]. In *BRCA1/2* mutated HR deficient tumors, unrepaired DNA causes cancer cell death and a favorable clinical response [11].

A significant clinical problem is the lack of effective treatment strategies for platinum-resistant ovarian cancer and *BRCA* wildtype HR proficient tumors that account for 49% of high-grade serous ovarian cancers [12]. Women diagnosed with HR proficient ovarian cancer have poorer clinical outcomes and fewer treatment options due in part to relative platinum and PARPi resistance [13–17]. The cyclin E1 gene (*CCNE1*) is commonly amplified or gained in ovarian cancer. *CCNE1* amplified tumors are characterized by aberrant replication, high levels of genomic instability, and replicative stress [13–19]. Because *CCNE1* amplified tumors are genomically unstable, they depend on intact BRCA1 for survival and are mutually exclusive to *BRCA1* mutant tumors. Therefore, *CCNE1* tumors are, by definition, HR proficient. Since *CCNE1* tumors account for ~20% of HR proficient HGSOC [17], *CCNE1* amplification alone may not be sufficient to confer relative resistance to platinum-based and PARPi chemotherapy.

Another newly discovered subtype of HR proficient high-grade serous ovarian cancer has amplifications in the bromodomain and extraterminal 4 gene (*BRD4*) and is similarly associated with poor outcomes [18,20]. BRD4 is a transcriptional regulator known to initiate transcriptional activity by stabilizing the binding of histones and transcription factors to form a bridge between promoters and enhancers [21]. BRD4 inhibition with bromodomain and extraterminal inhibitors (BETi) results in disassociation of promoters from enhancers, halting transcription and RNA synthesis. BRD4 has also been shown to be vital to DNA repair by stabilizing DNA repair complexes at double stranded breaks. Similarly, BETi treatment results in destabilization of DNA repair machinery, leading to accumulation of DNA breaks and ultimately cell death [21]. Thus, BETi could be incorporated in the treatment of HR proficient tumors. Indeed, our group and others have shown that treating *BRCA* wildtype HR proficient HGSOC with BETi, induces a HR deficient phenotype, sensitizes HR proficient tumors to PARPi and results in downregulation of cyclin E expression [22,23]. Targeting co-amplification of *CCNE1* and *BRD4* may be a useful treatment strategy for platinum and PARPi-resistant HR proficient HGSOC.

Prior publications have noted overlap of *CCNE1* and *BRD4* amplification in HR proficient HGSOC but have not characterized clinical outcomes for patients with both amplifications [18,22]. To identify patients who would benefit most from combination therapy with epigenetic drugs and PARPi more information is needed for *CCNE1* and *BRD4* concurrently amplified HGSOC. The main objective of this study is to test the hypothesis that HR proficient HGSOC with gene amplification of *CCNE1* and *BRD4* and elevated protein expression of both cyclin E and BRD4 will have worse poor prognosis than either amplification alone. The secondary objective is to determine whether protein expression by immunohistochemistry (IHC) of cyclin E and BRD4 can be used as prognostic biomarkers.

## 2. Methods

### 2.1. TCGA data

Published copy number data for *CCNE1* and *BRD4* and overall survival data were extracted from The Cancer Genome Atlas (TCGA) (www.cbioportal.org) provisional ovarian cancer dataset of 579 HGSOC. We used the provisional dataset as it included the largest cohort of patients with complete copy number variation and RPPA data. Amplified or non-amplified status was determined using GISTIC prediction by TCGA. OncoPrints were generated to demonstrate overlapping *CCNE1* and *BRD4* gene amplification in HGSOC. In addition, reverse phase protein array (RPPA) data for cyclin E and BRD4 protein expression was accessed for 482 HGSOC from TCGA through the Broad Firehose resource.

### 2.2. TMA data

Following IRB approval, a formalin-fixed, paraffin-embedded tissue microarray (TMA) was created from primary ovarian cancer samples from the Vanderbilt University Medical Center Tissue Repository for Ovarian Cancer (VUMC TROC) as previously described [24]. Of the 110 patients in the VUMC TROC TMA, the average age at diagnosis was 61 years, most patients were Non-Hispanic white with stage III or greater disease, 97 (88%) of patients underwent a debulking procedure, 27 (28%) had optimal debulking outcomes (defined as <0.5 cm at the time of data collection) and 54 patients (49%) had platinum sensitive disease (S1).

Immunohistochemistry (IHC) with anti-Cyclin E1 (Cat. ab33911, Abcam, Cambridge, MA) and anti-BRD4 (Cat. IHC-00396, Bethyl Laboratories, Inc., Montgomery, TX) was conducted by the Vanderbilt Translational Pathology Shared Resource. Whole-slide imaging and semi-quantitative measurement of the percentage of tumor cells showing positive expression was performed using the automated Ariol® SL-50 Platform in the VUMC Digital Histology Shared Resource (DHSR). For comparability with existing literature, IHC staining was first categorized as weak (<10% positive), moderate (10–50% positive), or strong (>50% positive). We also calculated an H-score (ranging from 0 to 300) by multiplying the percentage of cells staining positive (0–100) by the staining intensity (weak: 1, moderate: 2, or strong: 3) [25].

For response to chemotherapy, patients were classified as having platinum resistant disease by the clinical definition of recurrence within 6 months after completing platinum-based chemotherapy. Patients were classified as having platinum sensitive disease if they experienced recurrence more than 6 months following platinum-based chemotherapy or had no evidence of disease. Overall survival (OS) time was defined as the interval between ovarian cancer diagnosis and death, or else censored at last clinical contact. Progression free survival (PFS) time was defined as the interval between ovarian cancer diagnosis and disease recurrence, or else censored at death or last clinical contact.

### 2.3. Statistical analysis

Gene amplification of *CCNE1* and *BRD4* was correlated with protein expression levels in HGSOC represented in TCGA. Correlations between cyclin E and BRD4 protein expression and mRNA expression were quantified using Pearson’s correlation coefficients. For IHC analyses performed on the TMA, H-scores were dichotomized, with *high* defined as greater than or equal to the median value and *low* as less than the median value. Dichotomized variables for cyclin E and BRD4 protein expression were used to generated PFS and OS curves by Kaplan-Meier analysis and differences between curves were determined by log-rank tests. Age (continuous) and stage (early—stage I/II, late—stage III/IV) were included as covariables in Cox proportional hazards regression models to calculate Hazard Ratios (HR) and corresponding 95% confidence intervals (CI). Associations between cyclin E and BRD4 protein expression with platinum sensitivity were assessed by Mann-Whitney *U* tests. Survival curves were generated for patients with *CCNE1* amplification alone, *BRD4* amplification alone, both *CCNE1* and *BRD4* gene amplification, and no amplification in either gene. *P*-values ≤0.05 were statistically significant. All analyses were conducted with SAS v 9.4.

## 3. Results

### 3.1. CCNE1 and BRD4 are co-amplified with high protein expression levels in HGSOC

**Fig. 1.**
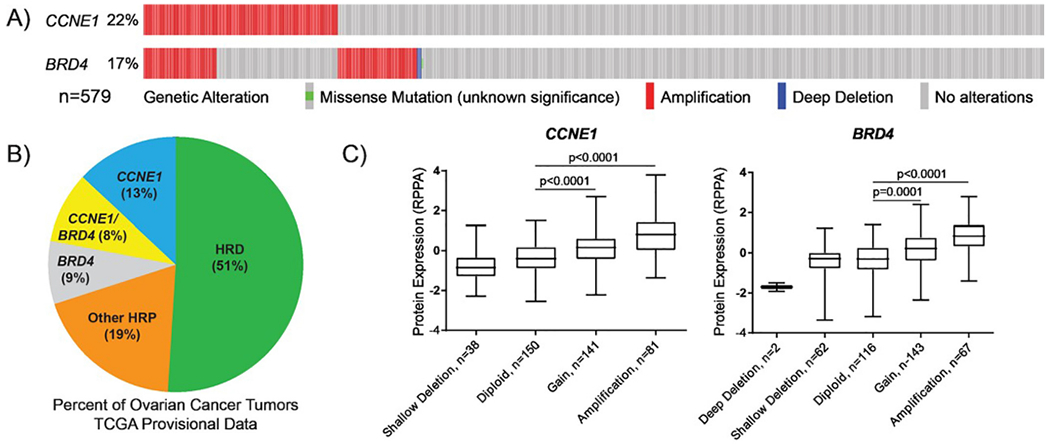
*CCNE1* and *BRD4* amplification and associations with cyclin E and BRD4 protein expression in HGSOC. A) *CCNE1* was amplified (red) in 22% and BRD4was amplified (red) in 17% of HGSOC. *CCNE1* was concurrently amplified in 47/98 (48%)with a *BRD4* amplification. *BRD4* was concurrently amplified in 47/125 (38%) with a *CCNE1* amplification. B) Percentage of TCGA HGSOC that were HR proficient (49%) versus HR deficient (51%) (adapted from ref. [17]. C) Cyclin E and BRD4 protein expression (by RPPA)was significantly elevated in copy number gain and copy number amplified compared to diploid tumors (two-way ANOVA) in HGSOC. (For interpretation of the references to color in this figure legend, the reader is referred to the web version of this article.)

Of 579 women with HGSOC from TCGA data, *CCNE1* was amplified in 125 (22%) and *BRD4* was amplified in 98 (17%). *CCNE1* amplification was detected in 48% (47/98) of HGSOC that harbored a *BRD4* amplification (Fig. 1A). A total of 176 (30%) HGSOC harbored an amplification in *CCNE1* and/or *BRD4* and 47 (8%) have both *CCNE1* and *BRD4* amplifications (Fig. 1B). Protein expression levels (as measured by RPPA) of cyclin E and BRD4 were increased in amplified and copy number gain HGSOC compared to diploid (Fig. 1C).

### 3.2. Cyclin E and BRD4 protein expression levels are positively correlated with each other by RPPA and IHC

**Fig. 2.**
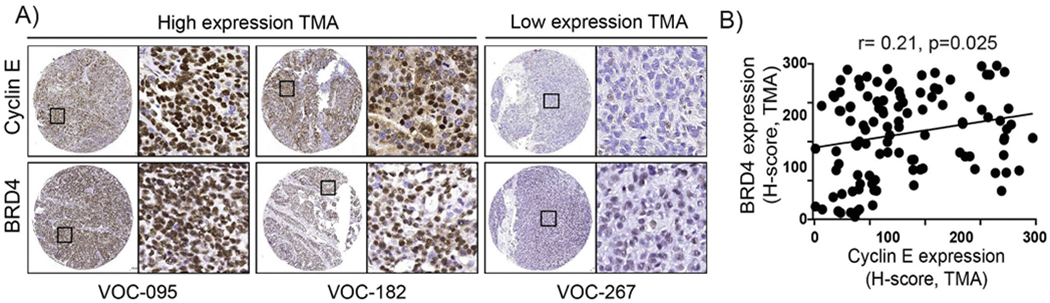
Cyclin E and BRD4 in ovarian cancer by IHC on a TMA. A) Representative high-grade serous ovarian tumors with high (H-score > 200) (left) and low (H-score <100) (right) expression levels of cyclin E and BRD4 by immunohistochemistry (IHC) on a tissue microarray (TMA). The inset shows a higher magnification image (×20) of each boxed area. B) Pearson correlation of H-scores for protein expression for cyclin E and BRD4 measured by IHC among 110 cases on a TMA (r = 0.21, *p* = 0.025).

To evaluate protein expression levels of cyclin E and BRD4 in TCGA HGSOC, RPPA data was assessed and revealed a positive correlation between cyclin E and BRD4 protein expression (S2, r = 0.23, *p* < 0.001). To evaluate protein expression levels of cyclin E and BRD4 by IHC, we assessed 110 high-grade serous epithelial ovarian cancer cases on a TMA from the VUMC TROC. Representative staining of cyclin E and BRD4 high and low expression are shown in (Fig. 2A). Consistent with TCGA data, cyclin E and BRD4 protein expression by IHC were also positively correlated in VUMC TROC HGSOC (Fig. 2B, r = 0.21, *p* = 0.025).

### 3.3. CCNE1 and BRD4 co-amplification and high protein expression of cyclin E are associated with poor clinical outcomes in patients with HGSOC

**Fig. 3.**
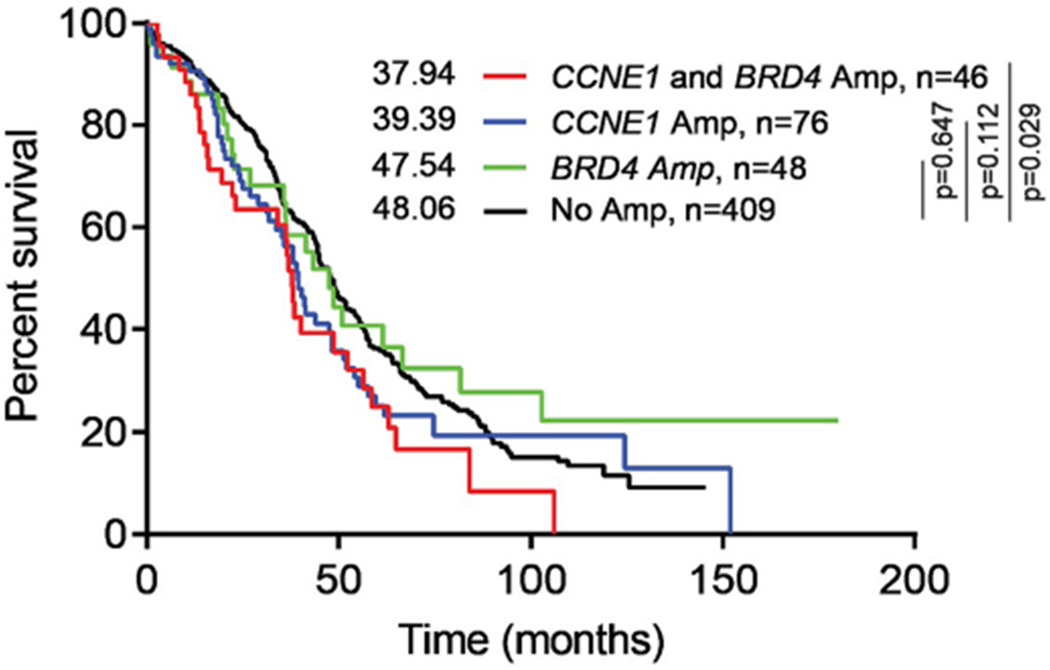
Co-amplification of *CCNE1* and *BRD4* is associated in poor overall survival. Kaplan-Meier analysis of HGSOC from TCGA provisional data (n = 579).

**Fig. 4.**
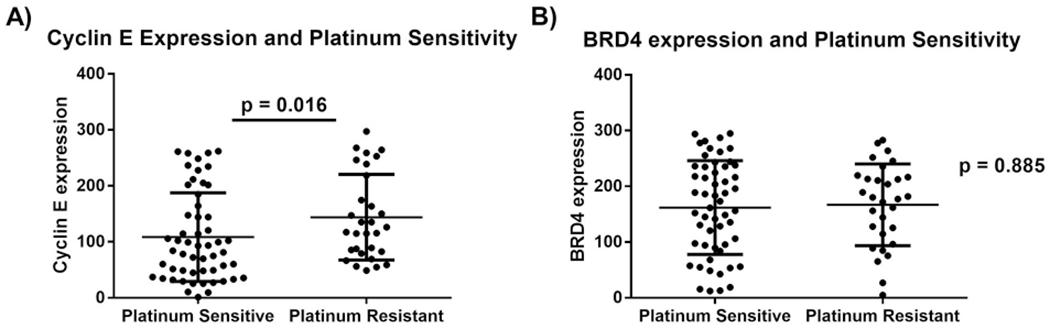
Cyclin E and BRD4 IHC expression in relation to response to platinum treatment. A) Cyclin E expression was increased in platinum resistant tumors compared to platinum sensitive patients (Mann-Whitney test, *p* = 0.016). B) BRD4 expression is not associated with platinum sensitivity (Mann-Whitney test, *p* = 0.885).

Patients with HGSOC containing both *CCNE1* and *BRD4* amplifications had shorter median survival (37.9 months) compared to patients without *CCNE1* or *BRD4* amplifications (48.1 months), *p* = 0.029 (Fig. 3). There was no significant difference in survival between patients who had only *CCNE1* amplification or only *BRD4* amplification compared to patients without either *CCNE1* or *BRD4* amplification, even after adjusting for age and stage of disease (Table 1). Among patients with HGSOC from the VUMC TROC TMA, high expression of Cyclin E by IHC was associated with worse overall survival, after adjusting for age and stage of disease (HR: 1.62, 95% CI: 1.04–2.53) (Table 2). BRD4 protein expression was not associated with overall survival. There was no difference in progression free survival for either protein. H-scores for protein expression were also stratified by platinum sensitivity. High expression of cyclin E was associated with platinum resistance (*p* = 0.016), while high expression of BRD4 was not (*p* = 0.885) (Fig. 4). The combination of high cyclin E and BRD4 expression was not associated with platinum sensitivity (Mann-Whitney test, *p* = 0.175, data not shown).

## 4. Discussion

In this study, we tested the hypothesis that HR proficient HGSOC with gene amplification and elevated protein expression of both cyclin E and BRD4 will have worse poor prognosis than either amplification alone. Of 30% of HGSOC with amplifications in *CCNE1* or *BRD4*, 8% have both *CCNE1* and *BRD4* amplification. This finding is consistent with frequent amplification of chromosome 19 in ovarian cancer, as *CCNE1* and *BRD4* are both located on chromosome 19: 19q12 and 19p13, respectively [18]. We found that clinical outcomes for HGSOC with both *CCNE1* and *BRD4* co-amplification have worse survival than patients without either amplification.

Prior studies using TCGA data have reported that HGSOC that harbor *CCNE1* amplifications are associated with shorter overall survival and resistance to platinum-based chemotherapy [17,18]. A recent study of TGCA provisional data by Aziz et al. showed that both *CCNE1* amplification and elevated cyclin E mRNA expression are associated with poor outcomes but *CCNE1* amplification alone or high cyclin E mRNA expression alone are not sufficient to drive poor survival [26]. Using the original published TCGA dataset [27], Zhang et al. showed that *BRD4* amplification is associated with poor progression-free and overall survival [28]. Similarly, Ucar and Lin [20], found *BRD4* amplification in 12% (57/489) of HGSOC of TCGA that was associated with worse overall and progression free survival. In that study, 26/57 (46%) of *BRD4* amplified cases also had a concurrent *CCNE1* amplification, similar to the 48% identified in our analysis of provisional TCGA data (Fig. 1A).

In contrast to prior studies, we compared survival of patients with only *CCNE1* or *BRD4* amplification alone, or patients with both, to patients without either *CCNE1* or *BRD4* amplification. Our results suggest that the poor prognosis previously reported in patients with *CCNE1* or *BRD4* amplification may not be due to either amplification alone but likely in combination with each other or possibly with amplification of other genes. Future studies will evaluate other gene amplification combinations.

Our second objective was to evaluate protein expression of cyclin E and BRD4 by IHC as potential prognostic markers. Of 110 patients, 32 (29%) had high expression of both cyclin E and BRD4 similar to the 30% of patients with amplification of *CCNE1* and/or *BRD4* in TCGA. However, our analysis of BRD4 and cyclin E protein expression by IHC did not indicate that survival was significantly worse for patients with high expression of both proteins. As recently shown by Aziz et al., high cyclin E protein expression without *CCNE1* amplification may not associated with poor outcome. Therefore, high cyclin E protein expression may not always correlate with *CCNE1* amplification. Because we do not have copy number data for the samples represented on the VUMC TROC TMA, *CCNE1* and *BRD4* amplification status in these HGSOC are not known. We also acknowledge the sample size is limited but believe these results provide the basis for future investigation for the prognostic relevance of CCNE1 and BRD4 co-amplification and overexpression.

Prior studies have shown that approximately 49% of HGSOC are HR proficient [12]. A significant problem is the lack of effective treatment strategies for HR proficient HGSOC known for poor clinical outcomes and decreased sensitivity to platinum-based and PARPi chemotherapy [13–17]. *CCNE1* and/or *BRD4* amplified HR proficient HGSOC (~30%) represent a substantial population for developing novel drug combinations. Our group and others have shown that in *BRCA* wildtype HR proficient tumors, epigenetic drugs including BETi, induce a HR deficient phenotype and sensitize tumors to PARPi [22,23,28,29]. PARPi and epigenetic drug combinations result in reduced HR proficiency, downregulation of cyclin E expression and increased DNA damage and apoptosis [22,29]. Thus, novel PARPi and epigenetic drug combinations may prove to be an effective strategy in platinum-resistant *CCNE1* and *BRD4* amplified tumors, a subset of poor prognostic HR proficient HGSOC.

Our study suggests that *CCNE1* and *BRD4* co-amplification might be more relevant to patient prognosis than either gene amplification alone. The high rate of amplification of *CCNE1* and/or *BRD4* (in 30% of HGSOC) suggests that targeting these tumors could be a useful strategy to extend PARPi combination therapies to women with platinum-resistant BRCA wildtype HR proficient HGSOC. Additionally, our study suggests that protein expression by IHC may not be a reliable surrogate marker for *CCNE1* and *BRD4* co-amplified HGSOC. Further studies are warranted to determine the use of fluorescent in situ hybridization (FISH) and/or genomic sequencing to identify *CCNE1* and *BRD4* co-amplified HGSOC.

## Figures and Tables

**Table 1:** TCGA copy number amplification and ovarian cancer survival outcomes. Ovarian cancer survival by *CCNE1* and *BRD4* copy number for 579 TCGA patients.

Gene	Median survival (months)	*p*-value	Hazard ratio and 95% CI^a^	*p*-value
No amplification	48.06	ref	ref	ref
*CCNE1*	39.39	0.129	1.23 (0.909–1.669)	0.176
*BRD4*	47.54	0.368	0.80 (0.527–1.222)	0.305
*CCNE1 & BRD4*	**37.94**	**0.044**	**1.48 (1.021–2.145)**	**0.038**

a Adjusted for Age (continuous) and Stage of Disease (early/late).

**Table 2:** IHC expression and ovarian cancer survival outcomes. Ovarian cancer survival (progression-free and overall) by Cyclin E and BRD4 protein expression for 110 HGSOC cases from the VUMC TROC.

Expression by IHC ^a^		Hazard ratio (HR) and 95% Confidence interval (CI)			
		
		Progression-free survival	*p*-value	Overall survival	*p*-value
Cyclin E					
	Unadjusted association	1.12 (0.74–1.68)	0.598	1.27 (0.83–1.97)	0.272
	Minimally adjusted ^b^	1.50 (0.98–2.28)	0.061	**1.62 (1.04–2.53)**	**0.033**
BRD4					
	Unadjusted association	0.99 (0.66–1.49)	0.959	0.87 (0.56–1.33)	0.511
	Minimally adjusted ^b^	1.14 (0.75–1.74)	0.551	0.99 (0.64–1.52)	0.954
BRD4 & Cyclin E					
	Unadjusted association	1.07 (0.68–1.69)	0.768	1.05 (0.65–1.68)	0.859
	Minimally adjusted ^b^	1.40 (0.88–2.25)	0.157	1.29 (0.86–1.99)	0.402

a Dichotomized by median H-score value, compared ≥ to < (reference).

b Adjusted for age (continuous) and stage of disease (early/late).

## HIGHLIGHTS

- Homologous recombination (HR) proficient high-grade serous ovarian cancers (HGSOC) are associated with poor clinical outcomes.
- About 30% of HGSOCs have amplifications in *CCNE1* and/or *BRD4* and 8% have co-amplifications in *CCNE1* and *BRD4*.
- *CCNE1* and *BRD4* amplifications are associated with increased protein expression in HGSOC.
- Elevated cyclin E protein expression by immunohistochemistry is associated with platinum resistance.
